# Surgery

**Published:** 1880-04

**Authors:** 


### SURGERY.

PARAPHIMOSIS—SIMPLE MODE OF REDUCTION.—(*Le Praticien*, No. 1, 1880.)

In very difficult cases, where ordinary means fail, Bardinet proceeds as follows: he takes a hair-pin, presses the points together somewhat, and inserts the curved end under the strangulation back of the gland. He then applies a second and a third at intervals around the gland; then, drawing the prepuce forwards, reduces it with great facility, the skin sliding over the three bridges without obstruction.

ERECTILE TUMOR TREATED BY INJECTION OF CHLORAL.—Dr. Antonio Pupi. (*Le Sperimentale* and *Le Praticien*, No. 62, 1879.)

A rapidly growing erectile tumor of the naso-palpebral region was injected at intervals of fifteen days with an aqueous solution of chloral, 1 to 10. After each injection, the consecutive tumefaction was almost immediate, but not painful, and only lasted four or five days. The cure was as complete as possible, scarcely any traces of the tumor being visible.

SPERMATIC COLIC.—M. Reliquet. (*Journ. de Méd.*, Dec., 1879, p. 552.)

The author reports the case of a man fifty years of age, in whom a diagnosis of tubercles of the prostate was made, and who presented the following symptoms; at first, sharp pains during coitus; painful excitation of the perineum, with frequent desire to urinate when riding in vehicles; on several occasions, brusque emission of a liquid analogous to the semen. The frequent desire to urinate became insupportable.

Rectal examination revealed a prostate with unequal lobes, the right presenting a well-defined protuberance prolonged backward, and continuous with the seminal vesicle. Pressure with the finger but slightly painful, but caused a desire to urinate. On introducing an elastic bougie, and pressing the tumor between the sound and the finger, a greyish mass escaped, resembling vermicelli, and the tumor diminished. Examination showed this to be composed of altered spermatozoa and mucus.

The spermatic colic, the retention of the semen in an altered ejaculatory canal, caused all the accidents. After repeated soundings, and further evacuations of this matter, the tumor disappeared. At the same time, the reflex trouble of micturition disappeared; even the bladder had been imperfectly emptied.

M. Reliquet has already noticed an analogous case, where the evacuation was caused by the presence of a lithoclast in the urethra.

He thinks, moreover, that real spermatic colic, less severe, exists especially in continent persons, which coitus relieves for a few days. The curative indications are very clear. One must understand this repletion to explain certain troubles of micturition, which otherwise remain inexplicable.

